# Macrophage Extracellular Traps: Current Opinions and the State of Research regarding Various Diseases

**DOI:** 10.1155/2022/7050807

**Published:** 2022-01-07

**Authors:** Weizhen Weng, Zuoyu Hu, Yunfeng Pan

**Affiliations:** Division of Rheumatology, Department of Internal Medicine, The Third Affiliated Hospital, Sun Yat-Sen University, Guangzhou 510630, China

## Abstract

Macrophages are an important component of the human immune system and play a key role in the immune response, which can protect the body against infection and regulate the development of tissue inflammation. Some studies found that macrophages can produce extracellular traps (ETs) under various conditions of stimulation. ETs are web-like structures that consist of proteins and DNA. ETs are thought to immobilize and kill microorganisms, as well as play an important role in tissue damage, inflammatory progression, and autoimmune diseases. In this review, the structure, identification, mechanism, and research progress of macrophage extracellular traps (METs) in related diseases are reviewed.

## 1. Introduction

Extracellular traps (ETs) are web-like structures composed of histones, double-stranded DNA, and elastases, which are ejected by immune cells and play a role in immune defense by capturing and killing bacteria, parasites, fungi, and other microorganisms. ETs were firstly described in detail in neutrophils as early as 2004 and named NETs (neutrophil extracellular traps), which can degrade virulence factors and kill bacteria [1]. Subsequent studies have shown that the process of immune cells forming ETs, known as “ETosis,” is morphologically and functionally distinct from other forms of programmed cell death and necrosis [2], since the initial reports of NETs and ETs have been found in a variety of other immune cells such as mast cells, eosinophils, basophils, monocytes, and macrophages. Meanwhile, ETs have been reported not only in humans or mammals (including cattle, horses, goats, and cats) but also in protozoans such as discoid amoeba and nonvertebrate such as arthropods, crustaceans, fish, birds, and plants [3, 4].

Macrophages are a group of immune cells that have virous roles in biology, from development, homeostasis, repair, immune response to pathogens, and source of inflammatory cytokines [5]. In 2010, it was reported for the first time that mature and differentiated macrophages can also produce ETs, called macrophage extracellular traps (METs). In this study, researchers found that mouse Raw 264.7 cell lines and mouse peritoneal macrophages could be stimulated to produce ETs by Staphylococcus aureus and phorbol-12-myristate-13-acetate (PMA) [6]. Another study has confirmed that METs can be produced by macrophages from different sources in response to a wide range of microorganisms and exogenous stimuli such as hypochlorous acid, PMA, IL-8, and TNF-*α* [7].

**Figure 1:**
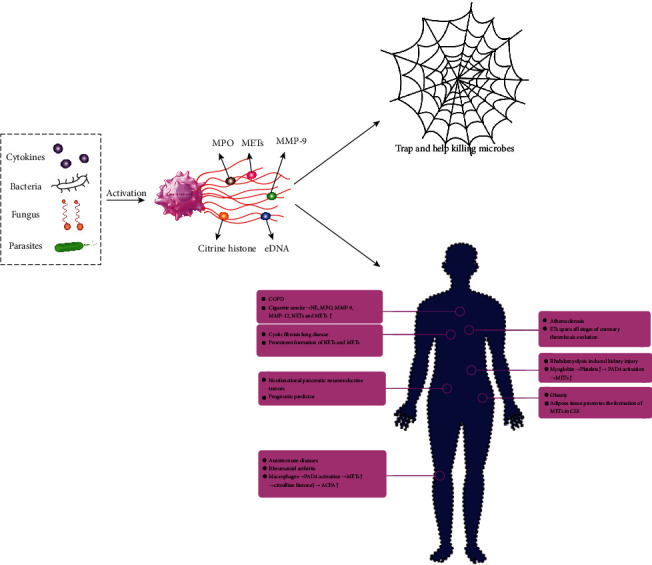
Macrophage extracellular traps (METs): induction, structure, and function. MPO: myeloperoxidase; MMP-9: matrix metalloproteinase-9; eDNA: extracellular DNA; COPD: chronic obstructive pulmonary disease; NE: neutrophil elastase; MMP-12: matrix metalloproteinase-12; NETs: neutrophil extracellular traps; PAD: peptidylarginine deiminase; ACPA: anti-citrullinated protein antibody; CLS: crown-like structure.

However, there are still not enough studies on METs, as the formation mechanism of METs and the relationship with diseases are not very clear. In this review, we briefly summarized the structure, identification, and mechanism of METs. Then, we focused on the current research progress of METs in a variety of diseases (Figure 1).

## 2. The Structure and Identification of METs

The structure of METs is similar to that of NETs in that they contain histones, double-stranded DNA, elastase, and myeloperoxidase. Some studies have confirmed that METs contain various components such as citrine histone [8–10], elastase [11, 12], myeloperoxidase [13, 14], matrix metalloproteinase-9 (MMP-9) [15], matrix metalloproteinase-12 (MMP-12) [16], CD68 [13], and lysozyme [17].

To identify METs, some studies use scanning electron microscopy to identify structures that originate from the macrophage cells [18, 19]. Furthermore, immunohistochemistry and immunofluorescence staining were used to stain and label the known components of the extracellular traps, observed by fluorescent microscopy and laser confocal microscopy [20]. To quantify METs, the proportion of METs formed in macrophage cells under multiple visual fields was monitored, and software such as ImageJ [21], Netquant [22], and DANA [23] was used for calculation. Some researchers also detected precise extracellular released DNA concentrations with SYTOX or PicoGreen reagent and kits read with a fluorescence plate reader [24, 25].

## 3. The Mechanism of MET Formation

Currently, there are few studies on the formation mechanism of METs. Considering the similar structures of METs and NETs, researchers focused on the formation mechanism of NETs, such as nicotinamide adenine dinucleotide phosphate (NADPH) oxidase/reactive oxygen species (ROS) system and peptidylarginine deiminase (PADS). Therefore, studies on kidney injury [8], COPD [15], aflatoxin B1 [12], Staphylococcus aureus [19], Streptococcus agalactiae [18], and Haemophilus influenzae [16] have shown that the formation of METs is related to the NADPH/ROS system. In these studies, attempts to inhibit ROS production by adding the NADPH oxidase inhibitor diphenyliodine (DPI) resulted in a reduction in MET formation.

However, other studies have also found MET production independent of the NADPH/ROS system, such as Candida albicans [26], Escherichia coli [17], and Mycobacterium tuberculosis [10, 11], since the addition of DPI did not hinder the formation of METs. Particularly, MET formation was found in Mycobacterium tuberculosis to be dependent on the virulence factor ESAT-6 or ESX-1 system. Other possible pathways of MET formation are statin-induced sterol pathway [6], tachyzoites of Neospora caninum-stimulated ERK1/2- and p38/MAPK-dependent cell death processes [14], and biochanin A-induced AMPK/ULK1/mTOR pathway [27].

Activation of PAD, through PAD4-mediated histone citrullination and nuclear chromatin depolymerization, has been shown to play an important role in the formation of NETs [28]. Obesity-induced adipose tissue inflammation might promote the formation of METs in CLS through PAD-mediated histone citrullination [29]. Additionally, macrophages might secrete functional PAD4 and release citrate histones through the formation of METS, inducing the production of ACPA and promoting the development of arthritis [9]. The formation of METs was also found to be related to PAD4-mediated histone citrullination in renal injury [8]. Interestingly, peripheral blood macrophages form METs when they are exposed to hypochlorous acid, PMA, IL-8, and TNF-*α* through a PAD-independent pathway, which was related to calcium influx [7].

More studies are needed to confirm whether the mechanism of extracellular trap formation is various according to different inducers, diseases, and environments.

## 4. The Study of METs in Disease

Studies on extracellular traps primarily focused on infectious diseases where in the early stage of infection, locally released chemotactic molecules attract and recruit innate immune cells such as neutrophils, monocyte, macrophages, and NK cells to phagocyte and kill the invading microorganisms. The formation of extracellular traps seems to serve as another defense mechanism by releasing granule proteins and chromatin, which together form extracellular fibers that bind Gram-positive and Gram-negative bacteria. These extracellular traps degrade virulence factors and kill bacteria [1]. Some studies have also found that extracellular traps can also capture and immobilize pathogens, preventing the spread of pathogens. On the other hand, METs might promote the survival of bacteria in host tissues through providing a scaffold for the aggregation of pathogens [17].

In addition, it has also been found that extracellular traps may promote the development of autoimmunity by generating persistent autoantigen-DNA complexes. Moreover, obstacles to the removal of extracellular trap components may lead to long-term exposure of autoantigens and promote the production of autoantibodies [30, 31].

Although most of the previously mentioned studies have focused on NETs, given that the structure of METs is similar to that of NETs, there have been many studies on the role of METs in infectious diseases and noninfectious diseases, which are summarized in Tables 1 and 2.

### 4.1. Infectious Diseases

#### 4.1.1. Gram-Positive Bacteria

Staphylococcus aureus is a Gram-positive bacterium that can cause many refractory nosocomial infections [32]. Shen et al. found that fosfomycin can promote the production of METs in murine peritoneal macrophages infected with S. aureus, which depends on the NADPH oxidase/ROS system, and meanwhile enhance the killing effect of macrophages against S. aureus [19]. Chow et al. found that statins could improve the ability of macrophages to kill Staphylococcus aureus by inducing the production of METs through inhibiting the sterol pathway in vitro and in vivo [6]. Streptococcus agalactiae, another Gram-positive bacteria, is associated with adverse pregnancy outcomes in pregnant women [33]. Doster et al. demonstrated that placental macrophages exposed to Streptococcus agalactiae in vitro could release METs and kill the organism, which depended on the production of ROS, and they found METs in human fetal membrane tissues infected in vitro. In addition, METs contained several matrix metalloproteinases that cause premature rupture of membranes. Thus, METs can respond to infection but also cause damage to the fetal membrane extracellular matrix [18]. Kalsum et al. confirmed that Mycobacterium tuberculosis could induce METs production in human macrophages, and this process was independent of ROS production but dependent on the virulence factor ESAT-6 [10]. Similarly, Wong and Jacobs reported that Mycobacterium tuberculosis can induce the production of METs in human macrophages, and the addition of IFN-*γ* can enhance the production of METs by promoting the ESX-1/RD1 protein secretion system [11].

### 4.2. Gram-Negative Bacteria

Liu et al. suggested that E. coli induced the formation of METs in mouse macrophages in a process independent of ROS produced by NADPH oxidase, and METs captured and killed E. coli at the infected site [17]. King et al. found that Haemophilus influenzae could induce the continuous production of ROS by human alveolar macrophages, which was related to the formation of METs and the expression of MMP-12 [16]. Zhao et al. found that biochanin A (BCA) could promote the release of METs through the AMPK/ULK1/mTOR pathway to clear extracellular Salmonella enterica. Furthermore, in vivo treatment with BCA increased intracellular and extracellular bactericidal activity by enhancing autophagy and MET production in peritoneal macrophages [27]. Similarly, a study conducted by Monaco et al. also indicated that Salmonella typhimurium induced METs released in murine macrophages. METs effectively immobilized and reduced Salmonella survival in a few minutes, suggesting METs as a novel immune-mediated defense mechanism against Salmonella infection [34].

### 4.3. Fungus

Previous studies have suggested that Candida albicans can activate neutrophils to induce the production of NETs, which in turn can capture and kill bacteria, but the antimicrobial efficacy of NETs is reduced in patients with neutrophil deficiency [35]. Loureiro et al. found that METs could be formed by macrophages in contact with Candida albicans, and this formation was proportional to the increase in multiplicity of infection. With the ability to capture and kill, Candida albicans can fight back by secreting DNases to degrade the main component of METs [26]. In the same context, Liu et al. found that Candida albicans could induce the formation of METs in mouse macrophages in a process independent of the NADPH oxidase/ROS system, but METs mainly inhibited the invasion of microorganisms by capturing them at the infected site, rather than directly killing them [17]. For mycotoxins, An et al. demonstrated that aflatoxin B1 induced the production of METs in a dose-dependent manner, and the formation of METs could reduce the content of aflatoxin B1, which was dependent on autophagy and the production of ROS [12].

### 4.4. Parasite

Bonne-Annee et al. found that human macrophages could be induced to produce METs by Strongyloides faecalis larvae in vitro, which captured and promoted the larval killing process. However, no METs were found in mouse macrophages in vitro, while the production of METs could be seen in the peritoneal exudate cells of mice [36]. Wei et al. showed that the tachyzoites of Neospora caninum could strongly induce the production of METs in bovine macrophages and trigger the formation of METs through ERK1/2- and p38MAPK-dependent cell death processes [14].

### 4.5. Leptospira

Nagel et al. found that both virulent L. interrogans sv Pomona strain AKRFB (P1) and its attenuated counterpart (P19) could lead to the production of METs in bovine macrophages [37].

## 5. Noninfectious Disease

### 5.1. Acute Kidney Injury

Rhabdomyolysis is a life-threatening disease caused by traumatic or nontraumatic muscle injury in which skeletal muscles break down and necrosis, resulting in myoglobin and other cellular proteins leaking into the circulation, leading to acute kidney injury. However, the mechanism is not yet understood [38]. In a mouse model of rhabdomyolysis induced by intramuscular injection of glycerin, Okubo et al. confirmed that heme-activated platelets released from necrotic muscle cells during rhabdomyolysis bind to macrophage antigen 1 (MAC1) to enhance the production of METs through increasing intracellular ROS generation and histone citrullination. In turn, this production contributes to the acute injury. METs were subsequently found in patients with rhabdomyolysis due to traumatic injury, with elevated free DNA levels in serum. To assess the therapeutic potential of targeting this pathway, the impact of lactoferrin (a glycoprotein that inhibits NETs) was investigated. They found that lactoferrin significantly inhibited MET formation and alleviated renal injury in glycerine-induced rhabdomyolysis mice. This study was the first to demonstrate that METs play a role in the pathogenesis of a disease, suggesting that the use of exogenous lactoferrin to inhibit the formation of MAC1 and METs is a potential therapeutic strategy for the prevention of rhabdomyolysis-induced acute kidney injury [8].

### 5.2. Atherosclerosis

Previous studies have shown that NETs are involved in human atherosclerotic plaques and thrombosis by promoting endothelial dysfunction, stimulating thrombosis, and stabilizing plaque formation [39, 40]. Pertiwi et al. found that METs were also present in atherosclerosis. By studying coronary atherosclerotic plaque in patients who died of acute myocardial infarction (AMI) in both fresh (representing recent-onset thrombus) and cell-rich organized masses (representing a thrombotic event several weeks ago), the study found that NETs dominated in early thrombosis and METs in late (organizing) thrombosis. Together, they spanned all stages of thrombus progression and maturation [13].

### 5.3. Chronic Obstructive Pulmonary Disease

Imbalance of proteases caused by smoking is a key process in the pathogenesis of emphysema [41]. The mechanism of this effect is not clear, but the formation of extracellular traps is related to protease expression and inflammation. King et al. have shown in vitro and in vivo that cigarette smoke significantly induces the formation of NETs and METs and simultaneously the expression of pathogenic proteases, neutrophilic elastase, MMP-9, and MMP-12, resulting in an imbalance of proteases leading to the occurrence and development of emphysema/COPD, which is associated with increased ROS production. The addition of DNase significantly reduced this response to cigarette smoke, as well as the number of macrophages and the degradation of lung proteins, suggesting a potential new therapeutic approach for COPD [15].

### 5.4. Cystic Fibrosis Lung Disease

Children with cystic fibrosis lung disease often have recurrent lower airway infections and prominent neutrophilic inflammation starting in the first year of life, resulting in persistent infection and inflammation leading to bronchiectasis [42]. In addition, neutrophil elastase activity in bronchoalveolar fluid is a major risk factor for bronchiectasis [43]. King et al. found prominent formation of NETs and METs in the bronchoalveolar fluid of children with cystic fibrosis lung disease that potentially led to lung injury. Furthermore, DNase 1 and *α*-1 antitrypsin may play a role in reducing lung inflammation in children. The possible combination of the two may be a new therapeutic strategy in cystic fibrosis lung disease [44].

### 5.5. Nonfunctional Pancreatic Neuroendocrine Tumors

At present, the clinical outcome prediction of nonfunctional pancreatic neuroendocrine tumors mainly depends on the WHO grade and TNM stage [45]. However, extracellular traps and macrophage infiltration can lead to disease progression, which are involved in the growth, proliferation, invasion, and metastasis of tumor cells [46]. Xu et al. indicated that the recurrence-free survival rate of patients with macrophage infiltration or METs positive in postoperative samples was lower, both of which were independent prognostic factors for recurrence-free survival and could be used as biological indicators for the prognosis of patients. Therefore, a new prognostic predictor composed of the WHO grade, TNM stage, and innate immune parameters was proposed, which can be utilized in the clinical application of chemotherapy and immunotherapy in nonfunctional pancreatic neuroendocrine tumors [47].

### 5.6. Obesity

Numerous studies in obese humans and animals have shown that macrophages infiltrate visceral adipose tissue and surrounding dead adipose cells to form the characteristic “crown-like structure” (CLS) morphology. Proinflammatory mediators produced by these immune cells are present in the peripheral blood of obese women and have been linked to the progression of breast cancer [48]. In the animal model of obesity, the occurrence of CLS is related to the activation of NF-*κ*B, and the increase in inflammatory mediators (such as TNF-*α* and IL-1*β*) is closely related to NETs and METs. Whether METs are formed in CLS remains to be confirmed [49]. To this question, Mohanan et al. found that macrophages produced METs after TNF-*α* stimulation in vitro and in obese mice, suggesting that obesity-induced adipose tissue inflammation promotes the formation of METs in CLS through PAD-mediated histone citrullination [29].

### 5.7. Autoimmune Disease

After the extracellular traps play their role in bacterial killing or tissue damage, they can be removed and degraded by macrophage phagocytosis and DNA enzymes. However, those that escape the clearance may generate a stable autoantigen-DNA complexes and lead to prolonged exposure of self-antigens, the generation of autoantibodies, thus promoting the occurrence and development of autoimmunity [50, 51]. There have been many studies on systemic lupus erythematosus (SLE), rheumatoid arthritis (RA), psoriasis, and autoimmune vasculitis related to NETs [30, 52–54]. NET formation is increased in patients with SLE. Neutrophils from these patients manifested phenotypic abnormalities such as enhanced aggregation and apoptosis. In RA, extracellular traps are considered a major source of citrullinated autoantigens. For example, NETs can be detected in synovial fluid and rheumatoid nodules in patients with RA, and serum levels of components of NETS in patients are higher than those in healthy controls [55]. However, there are few studies related to METs in autoimmune diseases. El Shikh et al. verified that macrophages could express functional PAD4 in murine collagen-induced arthritis (CIA) and synovial biopsies from RA patients. PAD4 that colocalized with lymphoid tissue peptidyl citrate could functionally deiminate extracellular proteins/peptides in vitro, release citrulline histone through the formation of METs, induce the production of ACPA, and promote the development of arthritis [9]. Since the components and structures of METs are similar to those of NETs, METs may also play a significant role in autoimmune diseases, which needs further researches.

## 6. Conclusion

ETosis is a process that is different from other programmed cell death and necrosis. Under various stimulation conditions, macrophages can produce fibrous network structures containing citrate histone, elastase, MPO, MMP-9, lysozyme, and other components. The existence and quantitative comparison of METs were identified by scanning electron microscopy, immunofluorescence, and other methods. The formation mechanism of METs has been found to be related to the NADPH oxidase/ROS system, as well as independent of the NADPH oxidase/ROS system. Under the stimulation of pathogens, macrophages induce the production of METs to remove pathogenic microorganisms such as bacteria, fungi, and parasites. Currently, most studies focus on the relationship between METs and infection. In recent years, studies have also found that METs play an important role in promoting tissue damage, inflammatory progression, and autoimmune diseases. More researches are needed in the future to deepen our knowledge and understanding of METs.

## Figures and Tables

**Table 1:** METs in infectious diseases.

Pathogens	Tissue	Cells	Inducers	Components of METs	Identification	Quantification	Mechanism of MET formation	Reference
Staphylococcus aureus		RAW 264.7 cells, murine peritoneal macrophages	Statins	H2A-H2B-DNA complexes DAPI	IF	Proportion of MET concentration of ET-DNA	Sterol pathway inhibition	[6]
		U937 cells						
Staphylococcus aureus		Murine peritoneal macrophages	Fosfomycin	Hoechst 33342, SYTOX	IF, SEM	Fluorescence intensity	NADPH/ROS system activation	[24]
		THP-1 cells						
Streptococcus agalactiae	Human placental	Human placental macrophages	Streptococcus agalactiae	Hoechst 33342, SYTOX, CitH3, MPO, NE	IF, IH, SEM	Proportion of METs	NADPH/ROS system activation	[25]
		THP-1 cells						
Escherichia coli		J774A.1 cells	Escherichia coli	Hoechst 33342, SYTOX	IF, SEM	Proportion of METs	Non-NADPH/ROS	[17]
		Murine peritoneal macrophages					System	
Haemophilus influenzae		BAL macrophages	Haemophilus influenzae	MMP-12	IF	Proportion of METs	NADPH/ROS system activation	[16]
Salmonella enterica		Raw 264.7 cells, THP-1 cells	Biochanin A	Hoechst 33342, SYTOX	IF	Fluorescence intensity	AMPK/ULK1/mTOR pathway activation	[27]
Salmonella typhimurium		J774A.1 cells murine macrophages	Salmonella typhimurium	Hoechst 33342, WGA	IF	Proportion of MET concentration of ET-DNA	—	[34]
Mycobacterium tuberculosis		Human macrophages	Mycobacterium tuberculosis	H4Cit3, DAPI	IF, SEM	Proportion of METs	ESAT-6 activation	[10]
Mycobacterium tuberculosis		Human macrophages	Mycobacterium tuberculosis	H4Cit3, Hoechst 33258, PicoGreen	IF, SEM	Proportion of METs	ESX-1 system activation	[11]
Candida albicans		Murine macrophages	Candida albicans	Candida albicans, SYTOX	IF, IHC	Proportion of MET fluorescence intensity	Non-NADPH/ROS system	[26]
		Murine peritoneal macrophages						
		J774A.1 cells						
Candida albicans		Murine peritoneal macrophages	Candida albicans	Hoechst 33342, SYTOX, histone, MPO, lysozyme	IF, SEM	Proportion of METs	Non-NADPH/ROS system	[17]
		J774A.1 cells						
Aspergillus		THP-1 cells	Aflatoxin B1	Hoechst 33342, SYTOX, MPO, NE, CitH3	IF	Fluorescence intensity	NADPH/ROS system activation	[12]
Strongyloides stercoralis		Human macrophages, murine	Larval Strongyloides stercoralis	Hoechst, MPO, histone 3	IF	Concentration of ET-DNA	—	[35]
		Macrophages						
Neospora caninum		Bovine macrophages	N. caninum tachyzoite	MPO, CitH3, SYTOX	IF, SEM	Concentration of ET-DNA	ERK1/2, p38/MAPK activation	[14]
Leptospira		Bovine macrophages	Leptospira	PI	IF	Concentration of ET-DNA		[36]

MPO: myeloperoxidase, NE: neutrophil elastase, MMP-12: matrix metalloproteinase-12, PI: propidium iodide; CitH3: citrulline histone 3; H4Cit3: citrulline histone 4; ET-DNA: extracellular DNA; BAL: bronchoalveolar lavage; SEM: scanning electron microscopy; IF: immunofluorescence; IHC: immunohistochemistry.

**Table 2:** MET in noninfectious diseases.

Diseases	Tissue	Cells	Inducers	Components of METs	Identification	Quantification	Mechanism of MET formation	Reference
Rhabdomyolysis-induced AKI	Renal tubules of mice	THP-1 cells	Heme-activated platelets	CitH3, Hoechst33342, SYTOX	IF	Proportion of METs	NADPH/ROS system activation PAD activation	[8]
Atherosclerosis	Coronary plaques	—	—	CD68, MPO, CitH3	IHC	Immunopositive cells per surface area	—	[13]
COPD	Lung tissue of mice	Alveolar macrophages of human and mice	Cigarette smoke extract	MMP-9, MMP-12, CitH3	IF, IHC	Tissue: cells per high-powered field Cells: proportion of METs Proportion of MET	NADPH/ROS system activation	[15]
Cystic fibrosis lung disease	Bronchoalveolar lavage fluid		Nontypeable Haemophilus influenzae	H3Cit, NE, MMP-9, chromatin	IF		—	[44]
Nonfunctional pNETs	Tumor tissue sections			H3Cit, MPO, CD68, DAPI	IF, IHC	Immunopositive cells per surface area	—	[47]
Obesity	Mammary gland adipose tissue of mice	RAW 264.7 cells	TNF-*α*	Phalloidin, DAPI, H4Cit3	IF, IHC	Fluorescence intensity	PAD activation	[29]
RA	Spleens and LNs of CIA mice	Splenic macrophages of mice	LPS	CitH3, GelRed	IF	—	PAD activation	[9]
	RA synovial tissues	RA synovial fluid macrophages						

MPO: myeloperoxidase; MMP-9: matrix metalloproteinase-9; CitH3: citrulline histone 3; H4Cit3: citrulline histone 4; TNF-*α*: tumor necrosis factor *α*; LPS: lipopolysaccharide; AKI: acute kidney injury; pNETs: pancreatic neuroendocrine tumors; COPD: chronic obstructive pulmonary disease; RA: rheumatoid arthritis; CIA: collagen-induced arthritis; IF: immunofluorescence; IHC: immunohistochemistry.

## Data Availability

No data were used to support this study.
